# Editorial Notes

**Published:** 1877-10

**Authors:** 


We welcome to the list of monthly medical periodicals the *New Orleans Medical and Surgical Journal*, which has hitherto been issued bi-monthly. Dr. S. M. Bemiss, formerly sole editor, has associated with himself Drs. W. H. Watkins and G. K. Pratt, as editors and proprietors. The first number as a monthly appeared for September. This will be a welcome change to the patrons of the Journal.

———:o:———

Lindsay & Blackiston are out with "Physician's Visiting List for 1878." They have been obliged to put it forth thus early in the season on account of the great demand for it. They also deserve much praise in that they have reduced the price of many of their books to 'suit the times.' We have received their classified list of recent and standard medical works which is worthy of the attention of the profession. It is to be had upon application.

———:o:———

Mr. J. H. Matteson, General Agent for Western New York, Western Pennsylvania, Michigan and Wisconsin, of Ziemssen's Cyclopædia of the Practice of Medicine, has removed his place of business from 271 to 365 Main St., at the bookstore of Herger & Ulbrich. If you have not already the numbers issued, go immediately and contract for them. The remaining numbers will be published at short intervals until the set is complete. Mr. Matteson is also agent for other standard medical works.

———:o:———

The Transactions of the Association of the Alumni and Officers of the Medical Department of the University of Buffalo, is now to be had upon application from Dr. E. N. Brush, 65 Ellicott St. Every graduate of the College should immediately send for one. The price of the volume in paper covers has been fixed at fifty cents, in boards at one dollar.

Napheys' Therapeutics.—Already the edition of this work which was published at the commencement of the present year is *entirely exhausted.* No higher testimony to its worth could be given. It recommends itself at once to every physician who sees it. As was remarked by a New York *Medical Record:* "As a handbook of Therapeutics, pure and simple, it is invaluable to every practicing physician ;" and the reason was well stated by the *American Medical Bi-weekly:* "In no work can the practitioner learn so easily as in this one the favorite medicines used in treating diseases, and the best methods of compounding them." A new edition (the *fifth*) is in active preparation.

